# The Critical Power Model as a Potential Tool for Anti-doping

**DOI:** 10.3389/fphys.2018.00643

**Published:** 2018-06-06

**Authors:** Michael J. Puchowicz, Eliran Mizelman, Assaf Yogev, Michael S. Koehle, Nathan E. Townsend, David C. Clarke

**Affiliations:** ^1^Department of Health Services, Arizona State University, Tempe, AZ, United States; ^2^Department of Biomedical Physiology and Kinesiology and Sports Analytics Group, Simon Fraser University, Burnaby, BC, Canada; ^3^School of Kinesiology, The University of British Columbia, Vancouver, BC, Canada; ^4^Division of Sport and Exercise Medicine, The University of British Columbia, Vancouver, BC, Canada; ^5^Athlete Health and Performance Research Centre, Aspetar Orthopaedic and Sports Medicine Hospital, Doha, Qatar; ^6^Canadian Sport Institute Pacific, Victoria, BC, Canada

**Keywords:** critical power model, W′ balance model, performance models, athletic performance, doping in sports, performance-enhancing substances, biomarkers, critical velocity

## Abstract

Existing doping detection strategies rely on direct and indirect biochemical measurement methods focused on detecting banned substances, their metabolites, or biomarkers related to their use. However, the goal of doping is to improve performance, and yet evidence from performance data is not considered by these strategies. The emergence of portable sensors for measuring exercise intensities and of player tracking technologies may enable the widespread collection of performance data. How these data should be used for doping detection is an open question. Herein, we review the basis by which performance models could be used for doping detection, followed by critically reviewing the potential of the critical power (CP) model as a prototypical performance model that could be used in this regard. Performance models are mathematical representations of performance data specific to the athlete. Some models feature parameters with physiological interpretations, changes to which may provide clues regarding the specific doping method. The CP model is a simple model of the power-duration curve and features two physiologically interpretable parameters, CP and W′. We argue that the CP model could be useful for doping detection mainly based on the predictable sensitivities of its parameters to ergogenic aids and other performance-enhancing interventions. However, our argument is counterbalanced by the existence of important limitations and unresolved questions that need to be addressed before the model is used for doping detection. We conclude by providing a simple worked example showing how it could be used and propose recommendations for its implementation.

## Introduction

Athletes have long used exogenous substances to enhance performance for personal gain (McHugh et al., 2005). In the past half-century, sporting federations created regulations and developed testing programs to detect and discourage this doping to create a level playing field. However, inconsistencies existed in how doping was addressed across sporting disciplines and regions; the World Anti-Doping Agency (WADA) was created in 1999 to harmonize these processes (Ljungqvist, 2014). The original strategy of doping detection was to detect evidence of banned substances by assaying biological fluids for illicit substances or their metabolites. While such direct detection methods have advantages, they are limited in important ways, especially for substances that are synthetic versions of naturally occurring endogenous hormones such as growth hormone (McHugh et al., 2005) and erythropoietin (EPO) (Pascual et al., 2004). Recently, indirect detection methods, which test for the biological effects of the substance rather than the substance itself, have shown both promise and limitations for detecting blood doping and exogenous EPO use. Accordingly, new approaches to anti-doping are needed. One strategy is to infer doping based on performance *per se*, which is sensible given that the within-subject coefficient of variation in performance is relatively low for elite athletes (Malcata and Hopkins, 2014), and ultimately, performance is the outcome that athletes are attempting to manipulate.

Herein, we review the potential for, and challenges of, applying the critical power (CP) model to anti-doping. The review is divided into five main sections. First, we discuss current biomarker-based doping control practices and their limitations. We then discuss in general terms the potential of performance-based markers as an additional class of evidence within indirect detection methods. We then narrow our focus to the CP model and describe in detail its basis and potential for doping control, followed by a detailed critical appraisal of its properties, which collectively show the model’s promise, limitations, and unanswered questions for this application. We conclude by offering guidelines for its implementation, recommending future research, and providing a simple worked example of its implementation. We also briefly review an extension of the CP model, the W′_bal_ model, which offers unique insight into performance during intermittent tasks. Because the CP model has yet to be scientifically evaluated in the context of doping control, our arguments are theoretical and supported by indirect evidence. We thus intend for the review to serve as a catalyst for discussion and to guide future studies in this area. Overall, we posit that the model holds promise for anti-doping, but gaps in knowledge and issues with the model must first be resolved.

## Existing Doping Control Practices

### The WADA Code: A Brief Overview

The first comprehensive list of prohibited substances (the World Anti-Doping Code) was released in 2004. Broadly, violations are grouped into *substances* and *methods* (World Anti-Doping Agency, 2017b): Substances are classified as (i) anabolic agents, (ii) peptide hormones, growth factors and related substances, (iii) β_2_ adrenergic receptor agonists, (iv) hormone and metabolic modulators, and (v) diuretics and masking agents. Additional substances that have not been approved for human therapeutic use are also prohibited, even if they are not listed. Three classes of prohibited methods exist: manipulation of blood and blood components, chemical and physical manipulation, and gene doping. According to WADA, a substance or method is prohibited if it meets two of the following three criteria: (1) it has the potential to enhance sport performance, (2) it represents a health risk to the athletes, or (3) it violates the spirit of sport (World Anti-Doping Agency, 2015). To enforce the Code, WADA conducts testing to detect doping, and the testing is either direct or indirect. Direct testing refers to the detection of a prohibited substance in a biological matrix such as blood or urine (Vernec, 2014). Indirect methods seek to detect the biological effects of doping rather than the substance or method itself; indirect methods have also demonstrated success by leading to sanctions in the absence of an adverse analytical finding (Vernec, 2014).

### Challenges With Enforcing the Code: Direct Detection

Directly detecting prohibited substances in athletes is challenging as demonstrated by the many athletes who passed doping tests throughout their careers only to belatedly confess to doping once retired (Vernec, 2014). Doping prevalence is estimated to be 14–39% of athletes, which far exceeds the 1–2% of annual sanctions for doping (de Hon et al., 2014). These estimates support the possibility that athletes may be successfully exploiting the time lag between the act of doping and its resultant detection window and the delayed but persistent performance benefit.

One example of a substance that is challenging to detect using direct methods is erythropoietin (EPO), a naturally occurring hormone that stimulates production of red blood cells by the bone marrow. Recombinant human EPO (rhEPO) was developed to treat anemia in clinical populations but has subsequently been used as an ergogenic aid due to its ability to increase hemoglobin mass, and hence oxygen carrying capacity of the blood (Clark et al., 2017). Despite its widespread use in the past (Thevis et al., 2017), its direct detection remains challenging because it is an analog of a naturally occurring substance in the body, it features high interindividual variability in athletes, and its levels change in response to various natural factors, including health, training load, altitude and even sleep apnea (Pascual et al., 2004).

For doping purposes, athletes may administer short-half-life rhEPO intravenously in more frequent smaller (‘micro’) doses than are used clinically. When taken in this manner, rhEPO is rapidly eliminated (Martin et al., 2016), such that a dose taken at night may be eliminated before the athlete is tested the following morning. Furthermore, rhEPO is typically used in the period prior to the competition because it has cumulative effects on hemoglobin mass that persist over the course of weeks (Clark et al., 2017), with the performance benefits possibly lasting longer. Therefore, the majority of EPO detection must occur through out-of-competition (OOC) testing, which consists of tests administered throughout the year, and which is logistically cumbersome in low-resource regions. Athletes have also employed strategies to mask the doping agent and its detectable metabolites, such as hyperhydration (Martin et al., 2016) or treatment of urine with proteases (Lamon et al., 2007), to dilute or degrade the compound prior to testing. Athletes are also permitted to miss two doping tests per year without triggering a sanction, which creates an additional loophole to circumvent OOC testing.

### Challenges With Enforcing the Code: Indirect Detection

The limitations of direct testing motivated the development of alternative indirect testing methods. The Athlete Biological Passport (ABP) is an example of an indirect testing method that has been effective in doping detection. It consists of two modules: hematological and steroidal. In the case of rhEPO detection and blood doping, the hematological profile is used to monitor several blood biomarkers known to be sensitive to blood manipulation (Sottas et al., 2006). By shifting from the detection of the stimulus to its hematological effects, the detection window is broadened and thus likely to better cover the period of performance enhancement. Furthermore, the ABP features a Bayesian model to determine an individualized expected range of normal values, which is updated over time based on the trends observed from longitudinal testing. Subsequent tests are compared to these ranges, and significant intra-individual deviations outside the individual’s normal range are flagged. This method improves the sensitivity and specificity of detection compared to using population norms. With this strategy, the ABP can be used to prompt direct targeted testing of athletes and to serve as evidence for establishing “Use” in pursuing a doping violation without having directly detected a prohibited substance or method (World Anti-Doping Agency, 2015).

The ABP features several limitations. First, it exhibits a lack of sensitivity to micro-dose rhEPO regimens that can raise hemoglobin mass by as much as 10% (Ashenden et al., 2011). Second, hemoglobin is measured as a concentration, such that the ABP can be subverted by hyperhydration (Bejder et al., 2016) and is compromised by natural plasma volume expansion during periods of heavy exercise load such as cycling grand tours (Corsetti et al., 2012). Third, concerns have been raised about the sensitivity, validity, and fairness of sanctions resulting from the ABP. Specifically, perturbations other than doping, such as altitude training, medications, bleeding ulcers, and bleeding hemorrhoids, can each cause blood parameter irregularities that could confound the ABP (Hailey, 2011). Lastly, the process by which the expert panel reviews suspect ABP results has been claimed to lack objectivity and transparency (Hailey, 2011). Additional strategies for doping detection are therefore sought.

## Performance as a Marker for Doping Detection

Since the primary goal of doping is to enhance performance, raw performance data, profiles, or derived metrics could serve as indirect markers of doping (Schumacher and Pottgiesser, 2009; Hopker et al., 2016). Indeed, the effectiveness of doping for enhancing performance has been shown by retrospective studies of professional cycling, which reported a period of rapid improvement in individual and group race speeds among top 10 finishers following the introduction of rHuEPO in the late 1980s (El Helou et al., 2010; Perneger, 2010; Lodewijkx and Brouwer, 2011) and a subsequent decline after 2004 as anti-doping efforts intensified (Perneger, 2010). Blood data from 2001 to 2009 corroborates suspected changes in doping behavior as elevated rates of abnormally high reticulocyte counts dropped after the introduction of the rHuEPO test in 2002, and the subsequent elevation of rates of abnormally low reticulocyte counts fell with the implementation of the ABP in 2008 (Zorzoli and Rossi, 2010). Similarly, improvements in group mean 5 and 10K running race speeds for the top 10, 20, and 40 performers, and the prevalence of “elite” and world-record individual performances have stagnated since 2005, coinciding with improved rHuEPO detection (Kruse et al., 2014). Hence, changes in performance coincided with trends in doping practices during these periods, such that performance may therefore serve as a marker for detecting doping.

Performance markers of doping offer several complementary advantages to biomarkers. First, performance enhancement manifests at the time of competition, whereas biomarkers may only be detectable in the weeks and months prior to competition when doping agents and methods tend to be used (USADA, 2012). Second, performance markers should be insensitive to practices used to subvert biologic detection protocols such as micro-dosing (Ashenden et al., 2011) and hyper-hydration masking (Russell et al., 2002), thus improving the sensitivity of testing. Third, statistical techniques for assessing time series data are well established (Shumway and Stoffer, 2017) and could be used along with data regarding typical errors of elite athlete performance, which tend to be relatively low compared to those of biomarkers (Hopkins et al., 2001; Bagger et al., 2003; Malcata and Hopkins, 2014). Hence, the underlying framework for an anti-doping performance test already exists, such that future developments in analytical approaches should be reasonably straightforward.

The feasibility of using performance markers for doping detection is clearest for sports such as track and field, weight lifting, and swimming in which the competition settings are relatively standardized, the outcome is a discrete, objective measurement of distance covered, mass lifted, or time achieved, and the athlete’s proficiency is highly correlated with specific physiological characteristics modifiable by doping agents. The relative standardization of the competition settings help minimize within-athlete variability (Malcata and Hopkins, 2014), such that results across competitions are directly comparable, and observed improvements in performance are likely due to improved physical capacity.

It is less evident how performance markers could be established for most other sports because the competition settings are less standardized and athlete physical capacity may not be the primary determinant of performance. For example, it would be less straightforward to detect suspicious performance of a soccer player. This gap may be addressable owing to the advent of player tracking technologies in which video systems or portable sensors are used to quantify player movements (Barris and Button, 2008; Aughey, 2011). From the changes in a player’s position over time, velocities and accelerations can be calculated (Aughey, 2011). In cycling, bicycle-mounted power meters enable the direct measurement of rider work intensity. The power or velocity data for each athlete can be summarized as a “mean maximal power (MMP) profile” or “record power profile” (Quod et al., 2010; Pinot and Grappe, 2011) or, equivalently, a mean maximal velocity profile (Delaney et al., 2015; Roecker et al., 2017). These profiles are predictive of future performances (Quod et al., 2010) and evolve as the athlete develops over time (Pinot and Grappe, 2015), such that unrealistic increases in the powers sustainable for the indicated durations could serve as evidence for doping.

Performance data nevertheless feature important limitations. The primary limitation is that performance data indicate what the athlete *did* rather than what *they were capable of doing*. Factors such as pacing, tactics, periodization, health, and environmental conditions will inevitably confound performance data. Another disadvantage is access to performance data. At the present time, athletes are not required to share their physiological or performance data, such that these data must be extracted from publicly available sources, which may be insufficient in terms of quality and quantity. The demand for data is particularly burdensome for generating an athlete’s MMP profile. Raw power data are needed for all workouts and competitions within the time frame of interest to ensure that the relevant best performances are captured (Quod et al., 2010; Pinot and Grappe, 2011, 2015). In addition, individual MMP data points do not predict performance at other durations. As a result, MMP profiles must feature sufficient sampling across all durations that may be of interest. Otherwise, comparisons cannot be made if future performances happen to occur for durations not already captured in the profile. Likewise, the MMP profile neither leverages neighboring MMP data points to reduce prediction errors nor features prediction intervals. Basing doping detection thresholds on MMP data alone would thus require population averages of performance variability, which would be wide compared to individualized prediction intervals. MMP data should therefore be supplemented with methods to interpolate performance at durations not included in the profile itself and to individualize the uncertainty estimates to the athlete being tested.

### Performance Models in Doping Detection

Performance models are mathematical representations of performance data and are useful for integrating data, inferring mechanistic parameters, and for predicting future performance. Examples of performance models include the CP model, which models the power-duration relationship, and the impulse-response model, which models the time course of performance as a function of daily training (Clarke and Skiba, 2013). The use of models may help to overcome the limitations of performance data, profiles, and simple metrics. In particular, performance models enable one to interpolate performances for values of the independent variable that were not originally tested. For example, the CP model reduces MMP data points to two parameters, CP and W′, which can then be used to predict performance for any duration within its domain of validity (Morton, 2006).

Importantly, metrics derived from performance models should in principle conform to the WADA code. According to WADA, “the ABP can be used to establish ‘Use’ per Code article 2.2 without necessarily relying on the detection of a particular Prohibited Substance or Prohibited Method” (World Anti-Doping Agency, 2017a). Additionally, the ABP is not specific to particular markers because both hematological (Sottas et al., 2009) and steroid profiles (Sottas et al., 2010) are now in routine use. Therefore, it is reasonable to suggest that indirect detection by athlete profiling is a general method and that performance-based markers should be acceptable under the WADA code. As such, performance metrics could form the basis of an *athlete performance profile*. The performance profile could then be used in a manner similar to the biological profiles in order to identify and target athletes for specific analytical testing, to pursue anti-doping rule violations in accordance with Article 2.2, to corroborate other analytical or non-analytical evidence (Saugy et al., 2014), or to monitor group prevalence (Sottas et al., 2011).

Like the WADA code for indirect testing, the Bayesian model underpinning the ABP is also general (Sottas et al., 2009, 2010). Detrended performance metrics could therefore be used as inputs to the Bayesian model and updated longitudinally at regular intervals to generate prediction intervals for the model parameters and its outputs. As one potential scenario, performance metrics could be combined with biological parameters similar to the OFF-hr score, which combines the concentrations of hemoglobin and % reticulocytes into a single score (Gore and Parisotto, 2003), or the abnormal blood profile (ABPS) score, which consists of seven hematologic parameters [red blood cell count, hemoglobin, hematocrit, mean corpuscular (MC) volume, MC hemoglobin, MC hemoglobin concentration, % reticulocytes] (Sottas et al., 2006). Alternatively, the Bayesian model of the ABP could be expanded for multiple lines of evidence. The current form of the Bayesian model features two variables, D and M, in which D is a binary variable that represents the state (doped or not doped) and M is a continuous variable that represents the biomarker. The causal relationship is specified as follows (Sottas et al., 2009):

$$P\left(D|M\right)=\frac{P\left(D|M\right).P\left(D\right)}{P\left(M\right)}$$

According to Bayes’ theorem, the model could be expanded as follows for multiple lines of evidence to find the probability of doping given both a biomarker (M_B_) and a performance marker (M*_P_*)

$$P\left(D|M_{B}\cap M_{P}\right)=\frac{P(M_{B}\cap M_{P}|D)\cdot P\left(D\right)}{P\left(M_{B}\cap M_{P}\right)}$$

Numerous models of the power (or velocity)-duration profile (“PD models”) have been proposed and are reviewed in detail elsewhere (e.g., Billat et al., 1999). Compared to other PD models, the CP model features several advantages for anti-doping applications. First, the CP model is the most extensively studied PD model (Morton, 2006; Poole et al., 2016) and has been validated for use with individual athlete data collected both in the lab and field (Skiba et al., 2014a; Karsten et al., 2015). Most other PD models have just been applied to world-record data (Billat et al., 1999). The CP model is also among the most parsimonious of the PD models, featuring just two adjustable parameters. Models with fewer parameters require less data for fitting. In the case of the CP model, only two performances at different durations are minimally required to estimate the model parameters. Finally, the CP model parameters are *physiologically interpretable*, and they change in predictable manners in response to physiological, nutritional, and ergogenic interventions. This feature is useful for doping detection because the parameters will change in a manner consistent with the mechanism of the doping method, which may thus provide insight into which doping method was used. In the remainder of the review, we discuss the suitability of the CP model for use in doping control.

## The CP Model: Basis, Physiology, and Current Applications

### Definition of the Model

**FIGURE 1:**
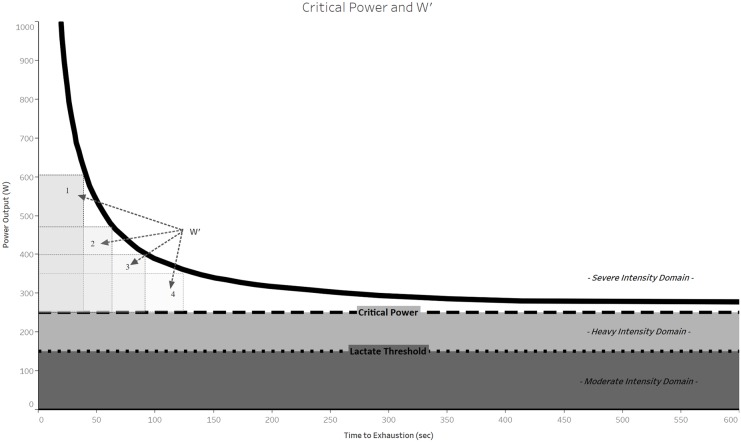
Schematic representation of the CP model of the power-duration relationship. Note the hyperbolic shape of the power-duration curve (thick black line) and that the four rectangles demarcating W′ have the same area, reflecting that W′ is a constant amount of work that can be done above CP.

A conserved hyperbolic relationship exists between maximally sustainable power output and duration (**Figure 1**). This relationship was first observed by Hill (1925) for world-record performances, followed by Monod and Scherrer (1965) for performance of isolated muscle groups (Monod and Scherrer, 1965), and then by Moritani et al. (1981) for whole-body exercise. Monod and Scherrer codified the CP concept into a two-parameter mathematical model:

$$t_{\lim}=\frac{a}{P-b}$$

in which *t_lim_* is the time to exhaustion, *P* is the power output during task performance, *b* is the asymptote of the curve, and *a* is curvature constant of the curve (Monod and Scherrer, 1965). Both *a* and *b* have physiological interpretations: *b* was called the “CP” and represents the power output that is sustainable for a very long time without fatigue (theoretically infinite time), and *a* is the total work that can be performed at intensities above CP.

The equation was subsequently restated by Moritani et al. (1981) in a linearized form:

$$P=\frac{W^{\prime }}{t}+CP$$

in which *P* = power output, *t* = time to exhaustion, *CP* is critical power (same as *b* in equation 1), and W′ is the work that can be performed above CP (same as *a* in equation 1) (**Figure 1**). Another common approach to expressing the CP model is to relate the total mechanical work done to CP and W′. This equation is also linear:

$$Work=CPt+W^{\prime }$$

Due to the difficulty of directly measuring mechanical power output for many exercise modalities, velocity is often substituted for power. The resulting *critical velocity model* features analogous parameters to those of the CP model: critical velocity (CV, units of distance over time) is used in place of CP and D′ in place of W′. D′ represents the distance that can be covered at intensities above CV.

The CP model permits clear physiological interpretations of the parameters but also requires several simplifying assumptions. Originally, CP was interpreted as the maximum power sustainable by steady-state aerobic energy provision whereas W′ was considered to represent the “anaerobic work capacity” (Moritani et al., 1981), which is defined as the mechanical work performed during exhausting exercise of sufficient duration to elicit near-maximal anaerobic ATP yield (Green, 1994). The assumptions are as follows: First, power output is assumed to be a function of energy generated from both aerobic and anaerobic pathways. Aerobic energy supply is not limited in capacity but rather by rate, and work done at or below CP is thus limited by the maximum rate of aerobic energy supply. Anaerobic energy supply cannot be sustained indefinitely and therefore W′ is assumed to be limited by capacity but not by rate (i.e., no limit to peak power or speed). W′ is defined as work done above CP to the limit of tolerance (Poole et al., 2016). When this limit is achieved, the sustainable power is markedly reduced (typically below CP), such that no more work above CP accumulates and a maximum value of W′ is thus achieved. The physiological interpretations of the CP model parameters enable the CP model to be used for assessing task-specific aerobic and anaerobic fitness.

Early investigations of the CP model in whole-body exercise suggested its benefits for athletic performance, based on its ability to define specific pacing strategies for continuous efforts (Moritani et al., 1981; Gaesser and Wilson, 1988). The model has since been extended to model intermittent performance (Morton and Billat, 2004; Skiba et al., 2012). Given that many sports are intermittent in nature, we discuss the suitability of the W′_bal_ model for doping detection in the final section of the review.

### Procedures for Estimating the Model Parameters for an Athlete

The most commonly applied CP test protocol requires the athlete to perform two or more time-to-exhaustion (TTE) tests. These tests consist of predetermined constant-work rates (CWR) that ensure the athlete will achieve exhaustion at particular durations. The athlete is typically granted 24 h or more of recovery between each test. The power (*y*-axis) vs. duration (*x*-axis) data from all trials is then fitted to the two-parameter CP model using ordinary least-squares regression. Early studies featured protocols consisting of two to seven trials to generate data for fitting the model (Hill, 1993). In addition, determining the powers for TTE/CWR tests is typically done using data from a graded exercise test, which requires an additional testing session. Therefore, CP testing using the TTE/CWR tests is time consuming, which limits the practical application of the method. Furthermore, these tests require control of the work rate, which is typically achieved using ergometers in laboratory-based settings.

To improve the time efficiency of CP estimation, Vanhatalo et al. (2007) proposed a new 3-min all-out test (3AOT) protocol conducted in a single testing session. This protocol is based on the assumption that a sufficiently long unpaced maximal effort (∼3 min) should fully deplete W′, such that the sustainable power beyond this time should be, by definition, equivalent to CP. Vanhatalo et al. (2007) validated the 3AOT against a traditional protocol consisting of TTE/CWR-based tests by showing that the end-test power from the 3AOT correlated with CP estimated from the traditional protocol [*r* = 0.99; standard error of the estimate (SEE) = 6 W] and the work completed above the end-test power correlated to W′ (*r* = 0.84; SEE = 2.8 kJ). The 3AOT has since been increasingly applied in research studies and in sport science practice as a time-efficient method to estimate CP and W′. However, its demanding nature is a disadvantage such that pacing is likely inevitable (Tsai, 2015) and the test also requires expensive laboratory-based cycle ergometers. Furthermore, several studies have reported that the end-test powers from the 3AOT likely overestimate the “true” CP (McClave et al., 2011; Bergstrom et al., 2013a,b; Nicolò et al., 2017).

Recently, time trials (TTs) and constant-duration tests have been increasingly used to estimate the CP model. In TT, the target distance or energy expenditure is determined and the athlete attempts to minimize the time to completion. In constant-duration tests, the trial duration is specified and the athlete attempts to maximize the average power or velocity over that time. The advantages of TT and constant-duration tests include lacking the need for a prior graded exercise test (which are used to determine the powers for TTE trials) and self-pacing may foster enhanced performance (Black et al., 2015) by enabling a fast-start strategy that results in faster 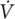
*O*_2_ kinetics (Black et al., 2015; Fullagar et al., 2016). Furthermore, TT and constant-duration tests can be conducted in the field using portable bicycle-mounted power meters, which enhances the feasibility and ecological validity of the CP model. Indeed, the need for an ecologically valid time-efficient protocol led Karsten et al. (2015) to evaluate a single-day field-based protocol for estimating the CP model. Their constant-duration protocol involved three trials of 12, 7, and 3 min in duration presented in this order and separated by 30 min of recovery. They compared this protocol to the conventional method of three TTE tests conducted in the laboratory. CP estimated from the two-parameter linear model was not statistically different between the two methods (mean difference = -2 ± 14 W; limits of agreement = -26 to 29 W) (Karsten et al., 2015). Similarly, W′ was not significantly different (mean difference = -0.14 ± 3.36 kJ; limits of agreement = -6 to 7 kJ) (Karsten et al., 2015). Similar results were obtained for CV modeling in running, as CV estimates from single-day field-based protocols featuring 30- and 60-min recoveries between the TT were not statistically different from those estimated using constant-velocity TTE tests, whereas the estimates for D′ were different (Galbraith et al., 2014). Hence, field-based, single-day protocols based on constant-duration tests can provide valid estimates for CP (or CV) but possibly not for W′ (D′). Indeed, estimates of W′ tend to be highly variable compared to those from TTE-based protocols (**Table 1**). The practical applicability of constant-duration trials would be further enhanced by minimizing the number of trials. A recent study compared CP and W′ estimates from protocols featuring either two or three constant-duration trials and found no difference in CP estimates (Parker Simpson and Kordi, 2016). These results corroborate those from earlier studies using TTE-based protocols, which showed that as few as two trials could be used to obtain accurate CP and W′ estimates (Hill, 1993). Therefore, two maximal-effort tests separated by as little as 30 min of recovery may represent an acceptable method for accurately modeling CP in the field. However, since the CP model has two adjustable parameters, a downside to protocols consisting of only two tests is that goodness-of-fit metrics and residuals cannot be computed.

**Table 1:** Critical power (CP) test protocol properties.

Property	CP test protocol				
	
	Constant work rate (power)/time to exhaustion (CWR/TTE)	3-min all-out	Constant-duration	Time trial (constant work or distance)	Field data (akin to constant-duration)
Independent variable	Power (ergometer) or velocity (treadmill)	Time (3 min)	Time (e.g., 3, 7, 12 min)	Measured times to completion for set work or distance trials	Time (e.g., 3, 7, 12 min)
Errors in IV^a^	Precision of power for cycle ergometers: 0.6–3.2% (Woods et al., 1994)	Negligible	Negligible	Ergometer – total work: precision should be similar to that of power	Negligible
	Accuracy of cycle ergometers: variable, often large systematic errors (∼10%) due to calibration, drift during the trial (Maxwell et al., 1998; Paton and Hopkins, 2001)			Distance: e.g., of a road course as measured by Jones counter or method of similar precision: ∼0.1%	
	Accuracy of treadmill velocity: one group observed accuracy within 0.02 m s^-1^ of desired speed (Galbraith et al., 2014)			(International Association of Athletics Federations [IAAF] and Association of International Marathons and Distance Races [AIMS], 2008; Georgopoulos et al., 2012)	
Dependent variable	TTE	Power vs. time curve	Mean power	Power (constant-work) or velocity (constant distance) calculated from the times to completion for the set work or distance trials	Highest average power^e^
Typical errors of the dependent variables of the test^b^	Cycling TTE (durations 2–20 min):	See typical errors of end-test powers (ETP) below	Mean power cycling, trial durations 2–60 min:	Mean power cycling, trial distances 5–20 km (∼6-25 min):	Typical errors for these types of data have yet to be published
	CV(%)^d^ = 10–19 (Hopkins et al., 2001; Currell and Jeukendrup, 2008)		CV(%) = 1.5-3.5 (Currell and Jeukendrup, 2008)	CV(%) = ∼1–2% (Hopkins et al., 2001; Currell and Jeukendrup, 2008)	Accuracy of on-board power meters, which can vary based on conditions (e.g., temperature): ∼ ± 2.5% (Paton and Hopkins, 2001; Gardner et al., 2004; Abbiss et al., 2009)
	TTE converted to power:		Mean velocity running, trial durations 2–60 min:	Mean velocity running, trial distances 1,500–5,000 m (∼6–20 min)	
	CV(%) = 1.5–2.7% (Hopkins et al., 2001)		CV(%) = 2.7 (Schabort et al., 1998)	CV(%) = 1–3% (Driller et al., 2017; Currell and Jeukendrup, 2008)	
	Running TTE: (durations 2–20 min)				
	CV(%) = 10 (Billat et al., 1994)				
	CV(%) = 13–15 (Laursen et al., 2007)				
Appropriate mathematical expression (based on the assigned independent and dependent variables)	Non-linear (equation 3)	CP = end-test power	Linear (equation 4)	Linear (equation 5, solved for t)	Linear (equation 4)
		(ETP) = mean power from final 30 s of the test			
		W′ = numerically integrated AUC of power vs. time curve bounded by ETP at bottom			
Typical errors in CP model parameter estimates^c^	Cycling:	Cycling:	Cycling:	Running:	Cycling:
	CP – CV(%) = 2–8%	CP – CV(%) = 1–7	CP – CV(%) = 2–3	Critical velocity – CV(%) = < 1–4%	CP – CV(%) = 3–4
	W′ – CV(%) = 7–14 (Hopkins et al., 2001)	W′ – CV(%) = 28 (Johnson et al., 2011; Wright et al., 2017)	W′ – CV(%) = 46 (Experiment 1, Karsten et al., 2015)	D′ – CV(%) = 9–18% (Galbraith et al., 2011, 2014; Nimmerichter et al., 2015)	W′ – CV(%) = 15–18 (Experiment 3, Karsten et al., 2015)
Pacing/variable power	No – constant, enforced by ergometer or treadmill	Theoretically no – maximum effort throughout; however, some pacing is likely	Yes	Yes	Yes
Time to complete test protocol	Hours (if trials on same day) to days	3 min for the test itself	Hours (if trials on same day) to days	Hours (if trials on same day) to days	Data collected over days-weeks


*^*a*^Errors in the independent variable reflect systematic and random error of the instrument and operator error. ^*b*^Errors in the dependent variable reflect the biological variability of performance, systematic and random errors of the instruments used to measure both the independent and dependent variables, and operator error. ^*c*^Errors in CP and W′ reflect the integration of errors propagated from the independent and dependent variables. ^*d*^CV(%) = coefficient of variation = standard error of the measurement/mean × 100. ^*e*^Highest average power outputs from field training, and racing/time trial data recorded by an onboard power meter.*

A final strategy for estimating the CP model is to extract mean-maximal power (MMP) profiles from power-meter data collected during all training and racing. MMP profiles are generated by extracting the highest average powers across a range of durations (Pinot and Grappe, 2011). Portions of these data can then be used to fit the CP model. CP models fit this way using MMP for 3, 7, and 12 min did not differ from models fit using laboratory-based constant-duration trials of the same durations (Fullagar et al., 2016). Similarly, CV models for athletes in timed sports such as swimming and running can be fit from race results over different distances (e.g., Dekerle et al., 2006; Jones and Vanhatalo, 2017). While convenient, CP models from race results can be confounded by issues such as the time between the sessions that led to the maximum powers for each duration, pacing and tactics, uncertainty as to whether maximal effort was applied, and environmental conditions.

### Physiological Interpretations

Monod and Scherrer (1965) originally described the CP of a muscle as corresponding to “the maximum rate it can keep up for a very long time without fatigue.” Thus, the physiological interpretation of both CP and W′ can be framed with reference to the mechanisms of fatigue. Accordingly, Poole et al. (2016) stated that “CP may be regarded as a ‘fatigue threshold’ in the sense that it separates exercise intensity domains within which the physiological responses to exercise can (<CP) or cannot (>CP) be stabilized.” Therefore, CP represents the highest intensity of exercise for which muscle metabolic homeostasis can be sustained. Since steady-state energy metabolism reflects matching between “wholly aerobic” energy supply and total energy demand, exercise performed at or below CP is not associated with rapid accumulation of fatigue inducing metabolites and is therefore sustainable for long duration. In contrast, exercise performed above CP requires a greater contribution of substrate-level phosphorylation to meet energy demand, which leads to a progressive depletion of PCr, increased [Pi] and [H^+^], decreasing metabolic efficiency, and continuously increasing 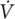
*O*_2_, until 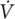
*O*_2max_ is attained (Grassi et al., 2015). Consequently, CP represents the boundary between achievable steady-state and non-steady-state aerobic metabolism, which corresponds to the heavy- and severe-intensity domains, respectively (**Figure 1**; Burnley and Jones, 2007). Many studies have sought to validate CP using physiological data. CP correlates with the power output at maximal lactate steady state (Pringle and Jones, 2002) and respiratory compensation point (Keir et al., 2015), both of which are classified as “second” or “anaerobic” thresholds (Binder et al., 2008). Furthermore, 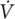
*O*_2_ achieves steady state for exercise at or below CP, but inexorably increases to 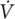
*O*_2max_ during exercise slightly above CP (Poole et al., 1988; De Lucas et al., 2013; Murgatroyd et al., 2014; Vanhatalo et al., 2016). In each study, participants achieved task failure markedly sooner for exercise slightly above CP.

W′ was originally considered to represent an energy reserve for mechanical work for power above CP (Monod and Scherrer, 1965). This energy reserve was thought to be from anaerobic sources (Moritani et al., 1981), such that W′ was subsequently conceptualized as a metric of *anaerobic work capacity* (Bulbulian et al., 1986; Nebelsick-Gullett et al., 1988; Housh et al., 1990). However, this terminology was deemed inappropriate for several reasons. First, the inexorable increase in 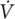
*O*_2_ until task failure means that oxidative phosphorylation contributes to the total energy supply for power above CP, such that W′ cannot be fully anaerobic in origin. Second, estimates of W′ were lower when modeled from trials performed in hyperoxia compared to normoxia (Vanhatalo et al., 2010a), suggesting that it is sensitive to oxygen availability and thus has an aerobic component. Third, after exhaustive exercise, the reconstitution of W′ is slower than the recovery of 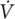
*O*_2_ but faster than lactate (Ferguson et al., 2010). This result implies that the kinetics of W′ reconstitution are not a unique function of phosphocreatine concentration, lactate concentration, or anaerobic energy *per se*. Lastly, it was found that skeletal muscle blood flow increases disproportionately during exercise above CP (Sarelius and Pohl, 2010). These authors concluded that increased muscle blood flow implies higher rates of oxidative metabolism, which is a characteristic of type-I muscle fibers. Hence, increased recruitment of type-I muscle fibers may help to protect against a progressive reduction in efficiency at or above CP (Murgatroyd and Wylde, 2011). Therefore, the three main mechanisms of energy production (PCr, glycolysis, oxidative) increase their energy output during exercise above CP and hence contribute to the energy store known as W′ (Grassi et al., 2015).

Although W′ is not uniquely determined by anaerobic capacity, it nevertheless correlates to various indices thereof, including to biochemical estimates from muscle biopsies (*r* = 0.73; Green et al., 1994), the mean power from the Wingate test (*r* = 0.74; Nebelsick-Gullett et al., 1988), accumulated work in high-intensity intervals (*r* = 0.74; Jenkins and Quigley, 1991), and maximal accumulated oxygen deficit (MAOD; W′ and MAOD were not different, Hill and Smith, 1993; *r* = 0.65, Muniz-Pumares et al., 2016). As discussed below, W′ is also sensitive to manipulations expected to change anaerobic capacity. Accordingly, anaerobic capacity is an important but not sole determinant of W′, such that W′ is potentially useful for detecting doping methods that seek to manipulate this capacity.

### Applications in Sport

The CP model has long been applied to analyzing and optimizing athletic performance. The model enables performance prediction, informs pacing tactics, and helps with the design of interval-training workouts (Pettitt, 2016). Furthermore, CP represents the boundary between heavy and severe-intensity exercise, such that it informs the training zones used by coaches in prescribing training intensity (Clarke and Skiba, 2013). The related W′_bal_ model enables the real-time monitoring of energy available for severe-intensity exercise, which could inform tactical decisions during competitions. The CP model has been used to derive insights into world-record performances (Dekerle et al., 2006). The model is applicable to diverse sports; it has previously been applied to individual sports such as cycling (Moritani et al., 1981; McClave et al., 2011; Karsten et al., 2015), running (Hughson et al., 1984; Hill et al., 2011), swimming (Wakayoshi et al., 1992; Toubekis and Tokmakidis, 2013), and rowing (Kennedy and Bell, 2000; Morton, 2009; Kendall et al., 2011), team sports such as rugby sevens (Clarke et al., 2014) and soccer (Clark et al., 2013) and racquet sports such as table tennis (Zagatto et al., 2008). The model has yet to be applied for doping detection, and this application would represent the most stringent test of its properties.

## Evaluation of the CP Model for Doping Detection: Promise and Challenges

The CP model could be used in three ways to suspect doping: (1) unrealistically high CP or W′ values compared to population norms, (2) unrealistic increase in one or both of the model parameters, CP or W′, within a given time frame, or (3) unrealistic performance compared to the prediction of an existing CP model within a given time frame. In each case, thresholds of suspicion must be established. These thresholds in turn would need to be based on scientifically justified abnormal values or rates of change that exceed the typical error of the measurement with high probability.

The severe consequences of doping sanctions on athletes, which include bans up to 4 years for first offenses and up to lifetime for second offenses, necessitates that any classification method used as evidence for sanctions must be highly *specific* for doping. The method must also be sufficiently *sensitive* to serve as a significant deterrent. Sensitivity and specificity are properties that express the ability of a continuous measurement to appropriately classify a subject in terms of a discrete feature or property; these properties are often visualized as receiver–operator characteristic (ROC) curves. Sensitivity is the true positive rate (dopers correctly classified as dopers) while specificity is the true negative rate (non-dopers correctly classified as non-dopers). In the case of CP-model-based doping detection, the continuous measurement would be the athlete’s CP, W′, or observed performance, which if outside a threshold value would classify the athlete as “suspected to be doping.” To be acceptable as a method for doping detection, a classifier based on the CP model would have to feature specificity greater than 99%, as required by WADA (World Anti-Doping Agency, 2014), and a sensitivity greater than the 10–20% estimated for existing detection methods (de Hon et al., 2014). The sensitivity and specificity of the CP model to classify dopers have yet to be scientifically studied.

Although no direct evidence yet exists pertaining to its properties as a classifier for doping detection, at least two indirect lines of evidence enable the evaluation of its potential for use in doping detection and to identify challenges to be resolved. These lines of evidence include (1) the sensitivity of the CP model parameters to performance-modifying manipulations and (2) the accuracies of the model parameter value estimates and the accuracy of the model predictions. In the discussion that follows, we employ the following definitions. *Accuracy* refers to the degree to which the estimate is different from the “true” value. It is analogous to *criterion validity*; however, we prefer “accuracy” rather than “validity” because of difficulties with interpreting the latter (Sechrest, 2005; cf. Newton and Shaw, 2013).

Additional concepts important to this discussion are reliability, minimally detectable change, and precision. Reliability is the reproducibility of the values measured in repeated trials conducted under the same conditions (Hopkins, 2000; Weir, 2005). Reliability is assessed through repeated measurements on the same subjects and is typically expressed in either relative or absolute terms. Relative reliability is expressed as the intraclass correlation coefficient and absolute reliability is expressed as the standard error of the measurement (SEM) (Weir, 2005). Absolute reliability is also commonly expressed as a coefficient of variation or *typical error*, which is the ratio of the SEM and the mean value of the repeated measures (Hopkins, 2000; Weir, 2005). Furthermore, the SEM determines the *minimally detectable change*, which is the smallest difference between measurements that can be considered real and not due to random error (Weir, 2005). Precision refers to the goodness-of-fit of a model to data, and is expressed as the R^2^ or model standard error of the estimate, and is reflected by the confidence intervals of the parameter estimates. While precision and reliability are not synonymous, reliability is intertwined with the precision of single measurements (Hopkins, 2000; Weir, 2005). Good precision and reliability are necessary for model accuracy.

Another important property is the typical variation in performance. While variation in athletic performance depends on the nature of the sport, the within-season coefficients of variation in race times across several sports are typically less than 2.5% (**Table 2**). Furthermore, within-athlete performance variabilities are similar across seasons; for example, skeleton, rowing, and cross-country skiing performance variations in race times were 0.5, 1, and 1.3%, respectively (Malcata and Hopkins, 2014). The potential usefulness of the CP model as a doping detection tool depends on its ability to detect performance gains beyond these predictable seasonal performance gains. Since the typical variations in performance tend to be small, and that these performance data are used to estimate the CP model, the typical errors of the CP model parameter estimates are likely to be small as well, as will their subsequent minimally detectable changes. The discussion that follows corroborates this expectation: the CP model is sensitive to the administration of performance-modifying substances and strategies.

**Table 2:** Examples of typical variation in race times for elite athletes.

Activity type	Distance/event	Season variation	Reference
Running	<3 km	Men: 0.8%	Hopkins, 2005
		Women: 1%	
Running	3–10 km	Men: 1.1%	Hopkins, 2005
		Women: 1.1%	
Track cycling individual pursuit	4 km	Men: 1%	Flyger, 2009
		Women: 1.2%	
Cycling road racing	Tour de France and World Cup (top eighth)	Men: 0.4–0.7%	Paton and Hopkins, 2006
Cycling time trials	Tour de France (top eighth) and International (top half)	Men: 1.3–1.7%	Paton and Hopkins, 2006
Triathlon	Olympic distance, total time for top-10% of finishers	Men: 1.1%	Paton and Hopkins, 2005
Mountain biking	World cups (top quarter)	Men: 2.4%	Paton and Hopkins, 2006
		Women: 2.5%	


### Sensitivity of CP and W′ to Performance-Modifying Manipulations

The CP model parameters are sensitive to performance-modifying manipulations, such as training, different environments, and ergogenic manipulations (**Table 3**). Importantly, CP and W′ tend to be sensitive to manipulations that are consistent with their physiological interpretations, which can provide clues as to the nature of the doping substance or method. Specifically, CP tends to be sensitive to substances and methods that improve oxygen transport whereas W′ tends to be sensitive to substances and methods that improve strength and power.

**Table 3:** Effects of performance-modifying interventions on CP model estimates.

Intervention	Dosage/exposure	Duration	Participants	Effect size^a^	Reference
Hypoxia	FiO_2_: 20% (∼250 m) vs. 12% (∼4,250 m)	Single exposure	9 trained male cyclists	CP: 2.98 ↓	Townsend et al., 2017
				W′: 1.19 ↓	
	FiO_2_: 21% (sea level) vs. 15.5% (∼2,500 m)	Single exposure	11 well-trained male cyclists	CP: 0.68↓	Shearman et al., 2016
				W′: 0.068 ↓	
Hyperoxia	FiO_2_: 70% vs. 21% (sea level)	Single exposure	7 habitually active males	CP: 0.77↑	Vanhatalo et al., 2010a
				W′: 0.81↓	
Caffeine	5 mg ⋅ kg^-1^ body mass	2 non-consecutive days	9 males	CP: 1.05↑	Silveira et al., 2017
				W′: 1.3↑	
	6 mg ⋅ kg^-1^ body mass	4 non-consecutive days	8 males	CP: 0.16↓	Moreira Gonalves et al., 2010
				W′: 0.8↑	
Creatine	20 g ⋅ day	5 consecutive days	8 healthy males	CP: 0.32 ↓	Miura et al., 1999
				W′: 0.98↑	
	20 g ⋅ day	5 consecutive days	10 physically active women	W′: 0.77↑	Eckerson et al., 2004
	20 g ⋅ day	5 consecutive days	19 participants	CP: 0.81↑	Jacobs et al., 1997
	20 g ⋅ day	5 consecutive days	15 untrained university students	CP: 0	Smith et al., 1998
				W′: 0.4↑	
	10 g ⋅ day	4 weeks	42 recreationally active men	CP: 0.26↑	Kendall et al., 2009
				W′: 0	
Bicarbonate	0.3 g⋅kg^-1^ body mass	5 consecutive days	8 trained male cyclists and triathletes	CP: 0.9↑	Mueller et al., 2013
	0.3 g ⋅ kg^-1^ body mass	Single trial	8 habitually active participants	CP: 0.06↑	Vanhatalo et al., 2010b
				W′: 0.11↓	
	0.3 g ⋅ kg^-1^ body mass	2 trials	11 trained cyclists	Normoxia	Deb et al., 2017
				W′: 0.4↑	
				Hypoxia	
				W′: 0.53↑	
“Pre-workout” supplement	10 g ⋅ day	3/per week /3 weeks	24 moderately trained recreational athletes	CV: 0.5↑	Smith et al., 2010
				W′: 0	
Erythropoietin	Meta–analysis of 17 laboratory studies	*n/a*		Aerobic performance:	Lodewijkx and Brouwer, 2011
				0.41–0.49 ↑	
Human Growth Hormone and Testosterone:	HGH: daily doses up to 30 μg ⋅ kg^-1^ body mass	12 weeks	14 middle-aged men	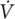 *O*_2max_: 0.76 ↑	Zając et al., 2014
	Testosterone: 100 mg; once a week			Anaerobic threshold: 0.68 ↑	
				Work rate max: 0.6 ↑	
				Total work: 0.29 ↑	
				Maximum power output: 0.27 ↑	
Ephedrine	0.8 mg ⋅ kg^-1^ body mass	Single day	10 males, 2 women	Time to completion: 0.43 ↓	Bell et al., 2002
	1 mg ⋅ kg^-1^ body mass	Single day	16 males	Power output 5 s Wingate test: 0.18↑	Bell et al., 2001
				Time to Exhaustion- MAOD: 0.35 ↑	
Training	Low intensity continuous exercise training/ high intensity interval training	6 weeks	14 males	CP: low intensity 1.8 ↑	Gaesser and Wilson, 1988
				High intensity: 2.5 ↑	
				W′: low intensity: 0.56↓	
				High intensity: 0.58↓	
	High intensity interval training	7 weeks	8 males	CP: 1.67 ?	Poole et al., 1990
				W′: 0.13 ?	
	High intensity interval training	8 weeks	19 males	CP: 0.56 ?	Jenkins and Quigley, 1993
				W′: 2.43 ?	
	Resistance training	6 weeks	16 males	CP: 0.87 ↓	Bishop and Jenkins, 1996
	High intensity interval training (with/without creatine supplementation)	6 weeks	42 active men	CP (Cr): 0.26 ↑	Kendall et al., 2009
				CP (Placebo): 0.165↑	
				W′ (Cr): 0.17 ↓	
				W′ (Placebo): 0.49 ↑	
	Resistance training	8 weeks	14 males	CP: 0.05 ↓	Sawyer et al., 2014
				W′: 1.02 ↑	


*^*a*^Effect size was calculated as Cohen’s d, whereby $ES=\frac{\overset{¯}{X_{1}}-\overset{¯}{X_{2}}}{SD}$ where $\overset{¯}{X_{i}}$ is the mean for the ith group and ${SD}_{pool}=\sqrt{\frac{S_{1}^{2}+S_{2}^{2}}{2}}$ where *S_*i*_* is the standard deviation for the ith group.*

#### Training

Critical power increases in response to both low-intensity continuous training (Gaesser and Wilson, 1988) and high-intensity interval training (Gaesser and Wilson, 1988; Poole et al., 1990; Jenkins and Quigley, 1993). Low-intensity, continuous training decreases W′ while the effects of high-intensity interval training on W′ remain controversial (**Table 3**). Resistance training reduces CP (Bishop and Jenkins, 1996; Sawyer et al., 2014) and improves W′ (Jenkins and Quigley, 1993; Sawyer et al., 2014).

#### Environmental Variables

Critical power increases with exposure to acute hyperoxia (70% O_2_, 30% N_2_) compared to normoxia, whereas W′ decreases (Vanhatalo et al., 2010a). The opposing responses of CP and W′ in this experiment may have been artifactually caused by the hyperbolic form of the model (see section below on “Model Bias and Artifacts”). In contrast, acute hypoxia treatment to simulate various altitudes decreases CP (Parker Simpson et al., 2014; Townsend et al., 2017) in a dose-response manner consistent with observed decrements in 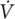
*O*_2max_ (Townsend et al., 2017). Specifically, CP decreased in proportion to simulated altitude, with significant reduction evident at 1,250 m. W′ was less sensitive to altitude change than CP as it was significantly reduced only at a simulated altitude of 4,250 m.

#### Ergogenic Aids

Ergogenic aids are substances or methods used to improve athletic performance. The effects of several ergogenic aids including caffeine, ephedrine, creatine, and bicarbonate have been tested for their effects on the CP model. An acute ingestion (60 min pre-workout) of caffeine (6 mg kg^-1^) significantly increased W′ (∼23%, effect size = 0.8) while CP was unchanged (Moreira Gonalves et al., 2010). However, a recent study found that a similar caffeine supplementation (5 mg kg^-1^, 60 min prior to the workout) significantly improved *both* W′ (effect size = 1.3) and CP (effect size = 1.5) (Silveira et al., 2017). Increases in both CP and W′ in response to experimental treatments are uncommon.

By comparison, acute ingestion of ephedrine (0.8 mg/kg) significantly decreased 10-km run times by approximately 48 s (Bell et al., 2002). Furthermore, ephedrine ingestion increased power output during the early phase of the Wingate test (effect size = 0.18), increased TTE (effect size = 0.35), and blood lactate, glucose, and catecholamine levels (Bell et al., 2001). Similar effect sizes were therefore observed for ephedrine intake and caffeine on performance measures reflecting aerobic and anaerobic fitness.

Creatine supplementation enhances the resynthesis of phosphocreatine (Williams and Branch, 1998), hence it is reasonable to expect that it might affect W′. Indeed, creatine supplementation (20 g for 5 days) significantly improved W′ [effect sizes = 0.98 and 0.74] (Miura et al., 1999; Eckerson et al., 2004). In contrast, the effect of creatine on CP is uncertain. Some studies have revealed small effects of creatine supplementation on CP (Jacobs et al., 1997; Smith et al., 1998), while creatine supplementation combined with high-intensity interval training was reported to significantly improve CP (Kendall et al., 2009). In the latter study, the duration of creatine supplementation exceeded those of previous studies by more than five fold (28 days vs. 5 days), which may explain the difference in the results.

Three types of bicarbonate supplementation protocols are typically employed: acute (single dose of ∼0.3 g⋅ kg^-1^ 60–90 min before competition), chronic (∼0.5 g⋅ kg^-1^ per day divided into 2–3 portions), and multi-day acute supplementation (one dose per day before competition for all days of the competition). A multi-day (5 days) acute bicarbonate supplementation in well-trained endurance athletes significantly increased W′ (effect size = 0.9) compared to placebo (Mueller et al., 2013). Acute bicarbonate supplementation (0.3 g⋅kg pre-exercise) did not affect W′ and CP in one study (Vanhatalo et al., 2010a) but significantly improved W′ in both hypoxic and normoxic environments (effect sizes = 0.4 and 0.53, respectively) in another study (Deb et al., 2017). This improvement was possibly due to enhanced buffering capacity that delays exercise-induced acidosis and enhances anaerobic energy supply (Deb et al., 2017).

Taken together, the effect sizes of performance-modifying treatments on CP and W′ are similar to those observed for doping agents (**Table 3**). These results therefore support the potential utility of the CP model for detecting doping in individuals. However, three caveats limit this claim. First, the study volunteers were not elite athletes and in some cases were untrained, such that the potencies of the ergogenic aids may be different than those observed in elite athletes. Second, the reported effect sizes of prohibited methods and substances are similar to those caused by legal performance-enhancing methods and substances, such that doping thresholds should exceed these effects to enhance detection specificity (i.e., avoid false positives). We note that anecdotally reported effects of doping typically exceed those reported in studies. Third, doping is always done in conjunction with other strategies to optimize performance, such that the observed changes to the model parameters in response to doping *per se* may be substantially less than those of isolated factors. At least one study examined the effects on CV of a supplement that contained several of the ergogenic aids listed above. Specifically, supplementation of participants with Game Time^®^ (Corr-Jensen Laboratories Inc., Aurora, CO, United States), which contains whey protein, cordyceps sinensis, creatine, citrulline, ginseng, and caffeine, was found to increase CV relative to placebo (+2.9%, effect size = 0.5) when combined with high-intensity interval training (Smith et al., 2010). Similarly, caffeine and ephedrine offered no additional benefit over ephedrine alone (Bell et al., 2001, 2002). The apparent lack of additive effects of performance-enhancing supplements reported in these studies suggest that higher sensitivity may be necessary to detect small additive or synergistic changes of prohibited agents on top of training effects.

### Accuracies of Model Parameter Estimates and Predictions of Performance

The accuracies of CP model parameter estimates and predictions of performance using the model represent a second line of indirect evidence for evaluating the potential of the model to detect dopers. Inaccurate models would be difficult to justify for use in anti-doping.

#### Accuracy of the Parameter Estimates

The accuracy of CP model parameter estimates is challenging to directly assess because there is no gold-standard measurement against which to compare them. In the past, the accuracy of the CP model was assessed according to its definition as the “maximal power that can be sustained without fatigue for a very long time” (theoretically infinite time). The accuracy of CP was accordingly assessed using TTE tests completed at CP, and CP was found to be sustainable for 20–60 min depending on the study (Hill, 1993; Vandewalle et al., 1997). The accuracy of CP was best when it was estimated from protocols featuring test durations that were well spaced in the domain of durations and that included a longer-duration test (e.g., >20 min; Vandewalle et al., 1997). Nevertheless, CP is inevitably inaccurate based on its original mathematical definition because the definition reflects the simplifying assumption that fatigue is solely caused by W′ depletion, which is physiologically untrue. Instead, assessing the accuracy of the CP estimates should be in light of its physiological definition, i.e., the maximum power at which muscle metabolic variables achieve steady state (Poole et al., 2016). To fulfill this criterion, participants should exercise at various powers near CP, during which measurements of physiological and metabolic variables are collected. Such studies (Poole et al., 1988; Jones et al., 2008; De Lucas et al., 2013; Murgatroyd et al., 2014) feature protocols in which exercise was performed at an intensity 5–10% above CP, the responses to which were compared to those of exercise at or slightly below CP. Steady states in physiological variables were achieved for exercise at or below CP but not for exercise above CP. The estimates of CP are therefore accurate at least to within 5–10% of the “true” physiological CP. These studies have typically featured specialized equipment that is inaccessible to most athletes; instead, emerging techniques such as portable near-infrared spectroscopy to measure muscle oxygenation may prove useful as a criterion measure.

As with CP, there is no gold-standard physiological measure of W′ that can be used to assess its accuracy. In the past, when W′ was conceptualized as the anaerobic work capacity, several groups tested the relationship between W′ and commonly used indirect measures of anaerobic capacity, such as Wingate tests and MAOD (discussed in the Section “Physiological Interpretations”). Subsequent studies showed higher correlations between W′ and MAOD when the W′ estimates had lower standard errors or when the estimates of W′ from the three common mathematical expressions of the two-parameter CP model (see equations 3, 4, and 5) were more similar (Hill and Smith, 1994). These precision criteria were then proposed as means to assure the accuracy of W′ estimates (Hill and Smith, 1994). However, the validity of this approach is limited because indirect measures of anaerobic capacity are themselves inaccurate. All indirect measures of anaerobic capacity are confounded by the contributions of aerobically produced energy and compromised by assumptions regarding efficiency of energy conversion (Green, 1994). The current definition of W′ is the mechanical work completed above CP until the limit of tolerance (Poole et al., 2016), and any future attempts to establish its accuracy must be in accordance with this definition.

#### Accuracy of the Model Predictions

While the accuracies of the parameter estimates are difficult to evaluate, the accuracy of performance prediction is more straightforward to evaluate because predicted performances can be compared to observed performances. For example, the CP model accurately predicted 2,000-m rowing-ergometer performance (Kennedy and Bell, 2000), and predicted marathon running performance better than 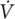
*O*_2max_ and ventilatory threshold (Florence and Weir, 1997). In general terms, the CP model is accurate for predicting performances when interpolated from within its domain of validity and is less accurate outside of that domain (Vandewalle et al., 1997), the reasons for which are described in more detail below.

#### Precision and Reliability

The precision of CP model fits to power-duration data tends to be excellent, with values of *R*^2^ typically well above 0.9. The typical errors of CP and W′ are respectively low and high (**Table 1**). An explanation for these observed typical errors is the hyperbolic relationship between power and duration: small increases in sustainable power at a given duration lead to large changes in TTE at the prior sustainable power. CP is relatively insensitive to errors in TTE, whereas W′ is highly sensitive to such errors (Vandewalle et al., 1997; see Figure 5 in that paper).

#### Model Bias and Artifacts

**FIGURE 2:**
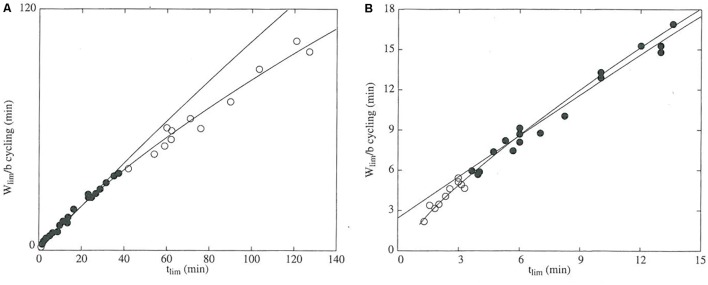
The domain of validity for the critical power model is defined by the durations of the exercise trials used to determine the power-duration relationship. The data were obtained from TTE trials for 10 participants, each of whom performed several trials at different power outputs. In both panels, the dependent variable is mechanical work accomplished in each trial (*W*_lim_) normalized to critical power (*b*) and is thus expressed in units of time (min). The independent variable is *t*_lim_, which is the duration of the trial (min). Black points denote data that were used to construct the regression line. **(A)** Data from all trials. The white points are data from TTE trials conducted at CP. **(B)** Data from the short-duration (0–15 min) trials. White points represent work accomplished during TTE trials lasting less than 3.5 min. Between the two plots, the times at which the white points deviate from the regression line indicate that the valid domain of the model ranges from approximately 3–40 min. The figure was reprinted by permission of Edizioni Minerva Medica from: *The Journal of sports medicine and physical fitness* (1997) 37, 89–102.

The assumed hyperbolic form of the power-duration curve introduces artifacts that bias estimates and predictions (Vandewalle et al., 1997). The departure of power-duration data from the hyperbolic curve is easily visualized (**Figure 2**) and demonstrates that the CP model will overpredict performance for trials whose durations are outside the range of those used in estimating the model (Pepper et al., 1992; Vandewalle et al., 1997). The physiological basis for this lack of fit is that numerous fatigue mechanisms operate to decrease sustainable power as duration increases (Burnley and Jones, 2007), whereas the CP model assumes that fatigue occurs solely during exercise above CP due to W′ depletion. The tendency for the CP model to overpredict performance represents a clear limitation of the CP model for doping detection. In addition, model lack-of-fit may manifest even within its valid domain, as non-uniformity of model residuals has been observed (Hinckson and Hopkins, 2005). The extent and implications of this lack-of-fit should encourage more authors to report residual diagnostics when using the CP model, which is standard procedure in statistical modeling for assessing model goodness-of-fit and validating the model assumptions (Morton and Hodgson, 1996).

Another possible artifact of the CP model is the anti-correlation of changes in CP and W′ in response to experimental treatments (Gaesser and Wilson, 1988; Jenkins and Quigley, 1993; Vanhatalo et al., 2010a; Poole et al., 2016). While the decrease in W′ might be real in some circumstances, at least two plausible explanations for this observation exist. First, the artifact might arise from the assumption of no rate limitation in W′ expenditure, which ignores the physiological reality that peak power is finite. This finite peak power may constrain improvements to short-duration performance in response to increased CP. In modeling improved CP, the hyperbolic function may compensate for these constraints by rotating counterclockwise, which results in reduced W′. Second, the artifact may result from learning effects affecting longer-duration TTE tests disproportionately compared to short-duration TTE tests (Hill, 1993). That is, learning effects may cause the study participants to improve more in the longer-duration tests than in the shorter duration ones over the course of repeated administrations of the tests. Improvements in the long-duration trials but not in the short-duration trials would artifactually increase CP and decrease W′. The impact of this potential anti-correlation artifact is unclear: on the one hand it points to a limitation of the model; on the other hand, simultaneous increases of CP and W′ may represent a potential standalone criterion for doping suspicion given that such changes are rarely observed in response to legal performance-enhancing strategies.

Finally, the precision and accuracy of CP model parameters and predictions are sensitive to the methods used for estimating the model. Several options are available for estimating the CP model, including the test protocol type (e.g., TTE, 3AOT, etc.; **Table 1**), the specific intensities or durations of the trials, and the mathematical expression used to fit the data (non-linear, linear power vs. inverse duration, linear work vs. time) (**Table 1**). The choice of the mathematical model depends in part on how the test was conducted and which variables are considered independent and dependent. For example, if TTE tests are used, the independent variable is the power and the dependent variable is duration. Conversely, if a TT is employed, then the distance is the independent variable and duration is the dependent variable. These assignments matter because the statistical procedures used to regress the variables feature assumptions about the errors of the variables. For linear regression, the independent variable is assumed to have no error; if it does, errors-in-variables models should be used because the estimates may be biased otherwise (Raboud, 2005). Hence, the common procedure of using the linearized form of the CP model for fitting TTE tests may lack statistical rigor, although the consequences of its use will depend on the magnitude of the errors in the TTE data.

#### Summary: Potential for and Limitations of the CP Model for Use in Doping Detection

Power-duration models are useful in anti-doping because of their ability to describe MMP data. Of existing power-duration models, the CP model holds particular promise for use in doping detection because its properties have been well studied, the model is simple (i.e., it features just two parameters) and thus requires relatively few data to estimate, and the physiological interpretations of the parameters mean that doping strategies will specifically enhance either CP or W′ depending on their mechanisms of action. From a statistical standpoint, the model can be used to detect doping if the doping effects cause changes to the CP model parameters or its performance predictions that exceed their typical errors and seasonal fluctuations due to legal performance enhancement strategies (e.g., training, ergogenic aids). The typical errors for CP are low, especially for constant-duration tests, TTs, and field data, while those for W′ are high (**Table 1**). The accuracy of CP estimates is also unknown but is at least within 5–10% of the “true” physiological CP. The accuracy of W′ estimates is doubtful given its large typical error. Hence, thresholds for detection for CP and predicted performance would be relatively narrow while the threshold for detection based on W′ would be relatively wide. Furthermore, the CP model is sufficiently sensitive to detect average changes in performance in response to treatments applied to groups of people and competitive performances of highly trained elite athletes are relatively invariant within and across seasons (**Table 2**). Accordingly, large increases in performance due to doping should be detectable using the CP model.

The promise of the CP model for anti-doping is counterbalanced by several limitations. A main limitation arises from the simplifying assumption that power-duration data are well described by a hyperbolic curve. Indeed, such data are well approximated by the curve within the domain of durations of the trials used to generate the data to estimate the model. Outside of that domain, the model will overpredict performance. A second limitation is the high typical error of W′ estimates. The importance of this limitation depends on the duration of the predicted performance because the relative influence of W′ decreases with duration as its contribution to total energy supply relative to CP diminishes. A third limitation is that the parameter estimates are sensitive to how the data were collected. The degree to which these limitations affect the ability of the model to detect doping is currently unknown.

## Implementing the CP Model in Doping Detection: Recommendations Regarding Methodology and Future Research

The preceding discussion motivates three methodological recommendations regarding implementing the CP model for doping detection. First, data of the highest quality should be used. Data for fitting CP models could come from several types of sources, such as from power or velocity data curated from athlete-monitoring devices or video tracking, or from publicly available databases of race results. It is also conceivable that the CP model estimates could come from laboratory-based testing. Regardless of the source, data from the same source should be used for longitudinal comparisons because of the sensitivity of the CP model to the test protocol and statistical procedure used to fit it. Furthermore, the limitations of a given data source must be acknowledged and explicitly accounted for. For example, field data from training and competitions represents what the person did and not necessarily what they were capable of doing, which could lead to artifactually large differences in CP and W′ estimates at different points in time. Second, the statistical procedure used to fit the model should suit the data source due to the potential bias that could be introduced if the independent variable has errors and the statistical procedure does not account for them. In addition, rigorous statistical procedure demands that the model residual diagnostic tests be performed for all model fits and confidence or prediction intervals be calculated for the model parameters and predicted performance. Finally, detection decisions must be insensitive to the consequences of the model’s simplifying assumptions. Power-duration data are well *approximated* by the hyperbolic function but lack-of-fit is to be expected. Detection thresholds must be sufficiently wide such that the lack-of-fit does not lead to false positives; however, wider detection thresholds reduce the sensitivity of the method.

The preceding discussion also revealed several open questions that must be resolved through new research. Most critically, the classification properties of the CP model (sensitivity, specificity) with regards to discriminating dopers and non-dopers must be characterized. The data showing the sensitivity of the CP model to individual treatments are insufficient in isolation because the model will have to detect performance enhancements due to doping that are inevitably confounded with those caused by legal performance enhancements due to training and use of ergogenic aids. A first study could involve applying the model to retrospective longitudinal velocity/power-duration data from a group of athletes that includes convicted dopers. A successful model-based doping detection approach should identify suspicious performances in the known doper cases.

Before such a study is possible, the method used to set the detection thresholds must be established. The simplest approach is to leverage existing statistical approaches for detecting changes in athletic performance (Hopkins, 2000, 2004; Bagger et al., 2003; Weir, 2005). These approaches have yet to be evaluated in their abilities to detect non-random changes in CP or W′ estimates. Even if these methods were found to be suitable, it would be best to integrate CP-model-based detection into the existing ABP framework because it is unclear whether doping sanctions could be assigned based on performance data alone. Instead, performance data should be included in the ABP as an additional independent source of evidence. How the CP model should be integrated into the ABP is currently unclear. The ABP works by monitoring biomarkers over time and comparing their values to thresholds that are set according to previously observed variation. The expected mean and variance of the biomarker is determined using a Bayesian approach in which an athlete’s values are first compared to a distribution generated from population norms and subsequently updated by the integration of the athlete’s own values. However, normative cross-sectional data are not yet available for the CP model, such that future studies employing meta-analyses of published data and cross-sectional studies of athlete populations are needed to estimate the population distributions of the CP model parameter values. Such studies should be supplemented with longitudinal monitoring studies of individuals to estimate the expected seasonal variations in CP and W′ (e.g., Passfield et al., 2017), which could further inform the prior distributions. Longer term studies of the trajectories of CP and W′ throughout athlete careers would provide information on the usual rates of improvement in these parameters as a function of athlete age and training experience. Suspicious rates of improvement could then be used as an additional variable for doping detection.

Finally, we recommend a third line of research in which the CP model statistical properties are studied. It would be beneficial to express the CP model as a formal statistical model that includes all the important sources of variance. Such a model would enable the study of how errors in the data collection propagate to the model parameter estimates, which in turn may enable the optimization of data collection protocols to minimize the uncertainties of the CP and W′ estimates. The data presented in **Table 1** could be useful for parameterizing such models.

## Implementing the CP Model in Doping Detection: an Illustration

We offer a simple example of how a CP-model-based detection method could be implemented. We extracted the data of Pinot and Grappe (2015), which features longitudinal MMP data from a professional grand tour cyclist, and added simulated doping effects for selected years. The dataset contains MMPs for each of the years from 2003 to 2008, of which the data corresponding to the 300, 600, 1,200, and 1,800 s durations were used to fit CP models, because these durations are within the valid domain of the CP model. In addition, the MMP data exhibited a log-linear increasing trend that is typical for a cyclist developing from age 18 to 23. For simplicity of this illustration, we removed this trend from the MMP values using log-linear regression so that we could fit the CP model to data from across multiple years in a manner similar to how the ABP is applied. In years 2007 and 2008, we increased by 5% all the MMP values to simulate “doped” performances while the MMP curves from 2003 to 2006 were left unchanged to represent baseline “clean” performances. In the absence of population norms for the CP model, we assumed that utilizing three or more years of baseline data (12 or more MMP data points) would generate individualized CP model thresholds comparable to a fully Bayesian implementation. This assumption is supported by previous work showing that *z*-score thresholds generated from an individual athlete’s data alone converge with the ABP model thresholds and demonstrate comparable classification performance once both models are trained on sufficient baseline data (Sottas et al., 2007). We thus chose to condition the CP model on years 2003–2005 to generate 99% prediction intervals that were used as the basis of comparison for the MMP data from 2006. Similarly, the data from years 2003–2006 were used to compare data from 2007, and data from years 2003–2007 were used to compare data from 2008.

**FIGURE 3:**
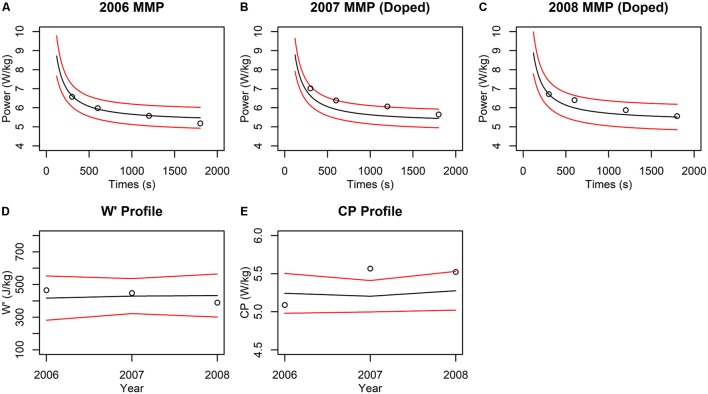
Example of the “CP Passport” for doping detection. **(A–C)** Scatter plots of MMP data (black circles) from the indicated year are plotted against the CP model predictions (black lines) and 99% prediction intervals (red lines) conditioned on the preceding years. Note that the MMP data for 2007 and 2008 contain simulated doping effects. Performances that exceed the upper red lines would be considered suspicious. **(D,E)** Scatter plots feature the estimated W′ or CP (**D,E**, respectively; black circles) plotted against the predicted parameter values (black lines) from the indicated year and 99% confidence intervals (red lines) conditioned on the MMP data from the preceding years. CP and W′ values in each plot were obtained by applying linear regression to the power vs. inverse duration data. The regression was computed using the “lm” function in R (version 3.4.0). The model curve and 99% prediction intervals were generated with the “predict” function. W′ and CP were estimated directly from the regression coefficients and 99% confidence intervals were estimated with the “confint” function. CP and W′ values that exceed the upper red lines would be flagged as suspicious.

We observed that the “clean” 2006 performances all fell within the prediction interval (**Figure 3A**), such that the performance would not evoke suspicion. In contrast, two out of four “doped” 2007 performances fell outside the intervals (**Figure 3B**), which would be classified as suspicious. Inspection of the parameter estimates revealed that the W′ and CP estimates for “clean” year 2006 both fell within the 99% interval, while the CP but not W′ fell outside the interval in the 2007 “doped” year (**Figures 3D,E**). The significant change in CP but not W′ can help predict the nature of the doping agent because changes to CP are consistent with the use of doping substances that increase oxygen transport but not muscular strength. Interestingly, neither the performances (**Figure 3C**) nor the parameter estimates (**Figures 3D,E**) for “doped” 2008 fell outside the prediction intervals. This result highlights a limitation in “passport-type” detection methods in which the “doped” 2007 data were included in the model training and biased the means and increased the variance such that the “doped” 2008 performances and parameter estimates were not statistically detected.

## Critical Review of the W′_bal_ Model

This review has focussed on utility of the CP model to doping detection; however, a limitation in the model’s utility is that performance predictions are for continuous exercise, whereas many sports are intermittent in nature. An important extension of the CP model is the “W′ balance” or “work-balance” (W′_bal_) model, which predicts W′ levels over time during intermittent exercise featuring bouts of severe-intensity exercise alternated with bouts of recovery (Skiba et al., 2012). This model offers unique information for doping detection that complements that of the CP model, such that it deserves discussion.

The empirically derived W′_bal_ model stipulates that the remaining amount of W′, or “*balance*” of W′ (W′_bal_), is the total W′ (W′_o_) subtracted by the product of W′ expended in a prior bout of severe-intensity exercise (W′_exp_) and a decreasing exponential function of time, with time constant, τ_w′_:

$$W_{bal}^{\prime }=W_{0}^{\prime }-\int_{u}^{t}W_{\exp}^{\prime }e^{-\left(\frac{t-u}{\tau_{w^{\prime }}}\right)}dt$$

$$\tau_{w^{\prime }}={{564e}^{-0.01D}}_{CP}+316$$

where *D*_CP_ is the difference between CP and the power during the recovery. The decreasing exponential diminishes to zero in time, causing its product with W′_exp_ to decline as well, such that this function models the recovery of W′_bal_ to W′_o_. To determine the nature of τ_w′_, the model was used to simulate W′_bal_ in response to four protocols involving intermittent interval exercise to exhaustion. The four protocols featured 60-s severe-intensity exercise alternating with 30-s of recovery exercise, the intensity of which was different for each protocol (20 W, moderate, heavy, and severe intensities). τ_w′_ was then fit to cause the modeled W′ to equal zero when the subject was exhausted. τ_w′_ was observed to increase (i.e., recovery took longer) as a function of the recovery intensity. Subsequent studies further revealed the sensitivity of τ_w′_ to work and recovery bout durations (Skiba et al., 2014a) and to environmental conditions (Townsend et al., 2017).

The W′_bal_ model adds unique information that could be used as evidence for doping detection. First, the model is useful for describing stochastic exercise featuring bouts of intermittent high- and low-intensity exercise, such as could be expected in tactical races and field-based sports. Since the point of exhaustion during a maximal bout of intermittent exercise should theoretically coincide with complete expenditure of W′ (i.e., W′_bal_ has reached 0 kJ), suspicion of doping might arise from W′_bal_ repeatedly declining well below zero and the lower bound of the model’s prediction interval, which would be physiologically implausible. Alternatively, it is possible that unrealistically high values for τ_w′_ are observed, which would signify implausibly fast recovery kinetics from high-intensity efforts.

The promise is counterbalanced by issues regarding the accuracy of the model and its sensitivity to different conditions. First, the model takes as input W′, which is difficult to accurately estimate as discussed above. Furthermore, τ_w′_ is determined in part by CP, and both CP and W′ are sensitive to environmental conditions such as altitude (Shearman et al., 2016; Townsend et al., 2017), which serve to reduce the accuracy of the W′_bal_ model if such factors are left unaccounted for. In addition, the depletion and recovery kinetics are dependent on task features such as work and rest durations (Skiba et al., 2012, 2014b) and pacing strategy. While the W′_bal_ model might be suitable for predicting when a rider is approaching exhaustion (Skiba et al., 2014a), it is questionable as to whether it can provide quantitative predictions of athlete performance capabilities with the stringency required for doping detection. Future research is recommended that focuses on modifying the W′_bal_ model in a manner that improves its accuracy for predicting performances across a broad range of intermittent exercise protocols.

## Conclusion

Herein we reviewed the potential of the CP model for use in anti-doping. We conclude that the model, or improved versions thereof, hold promise for doping detection because of its sensitivity to performance-enhancing manipulations, its physiological interpretability, the low data burden, and its suitability for use in established statistical frameworks for monitoring of individuals and/or the ABP. We caution, however, that important limitations exist in applying CP-model-based doping detection. First, the discriminative abilities of the CP model for classifying dopers and non-dopers has yet to be directly studied and only indirect evidence is available to support its use. Second, the simplifying assumption of the hyperbolic function for approximating the power-duration relationship introduces biases and artifacts that reduce the model’s accuracy. Third, the model parameter estimates are sensitive to the source of the data and the data quality; W′ estimates are especially imprecise. Given the severe consequences of a positive doping test, these limitations ought to be addressed prior to the adoption of the CP model as an anti-doping tool.

## Author Contributions

NT and DC conceived the work. MP, EM, AY, MK, NT, and DC each contributed sections of the manuscript and contributed to editing drafts of the full manuscript. DC oversaw the project. All authors approved the final submission.

## Conflict of Interest Statement

The authors declare that the research was conducted in the absence of any commercial or financial relationships that could be construed as a potential conflict of interest.
